# Direct arylation and heterogeneous catalysis; ever the twain shall meet

**DOI:** 10.1039/c5sc01534k

**Published:** 2015-06-19

**Authors:** Rafael Cano, Alexander F. Schmidt, Gerard P. McGlacken

**Affiliations:** a Department of Chemistry , University College Cork , Cork , Ireland . Email: g.mcglacken@ucc.ie; b Analytical and Biological Chemistry Research Facility , University College Cork , Cork , Ireland; c Faculty of Chemistry , Irkutsk State University , Irkutsk , 664033 , Russia . Email: aschmidt@chem.isu.ru

## Abstract

The formation of aryl–aryl bonds and heteroaryl analogues is one of the most important C–C bond forming processes in organic chemistry. Recently, a methodology termed Direct Arylation (DA) has emerged as an attractive alternative to traditional cross-coupling reactions (Suzuki–Miyaura, Stille, Negishi, *etc.*). A parallel focus of the pharmaceutical and other chemical industries has been on the use heterogeneous catalysis as a favourable substitute for its homogeneous counterpart in cross-coupling reactions. Only very recently has heterogeneous catalysis been proposed and applied, to DA reactions. In this perspective, we consider the terms ‘heterogeneous’ and ‘homogeneous’ and the problems associated with their delineation in transition-metal catalysed reactions. We highlight the reports at the interface of DA and heterogeneous catalysis and we comment briefly on the methods used which attempt to classify reaction types as homo- or heterogeneous. In future work we recommend an emphasis be placed on kinetic methods which provide an excellent platform for analysis. In addition two analytical techniques are described which if developed to run *in situ* with DA reactions would illuminate our understanding of the catalysis. Overall, we provide an entry point, and bring together the mature, yet poorly-understood, subject of heterogeneous catalysis with the rapidly expanding area of DA, with a view towards the acceleration of catalyst design and the understanding of catalyst behaviour.

## Abstract 2

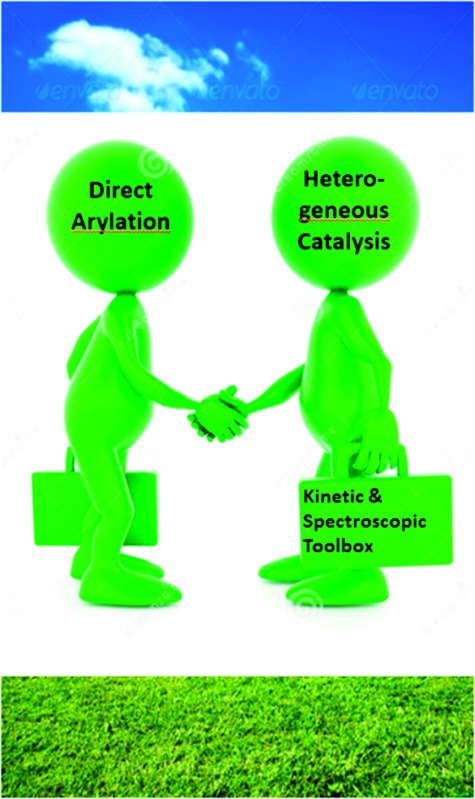
We bring together the mature, yet poorly-understood, subject of heterogeneous catalysis with the rapidly expanding area of Direct Arylation, with a view towards the acceleration of catalyst design and the understanding of catalyst behaviour.

## Introduction

### Direct arylation

**Fig. 1:**
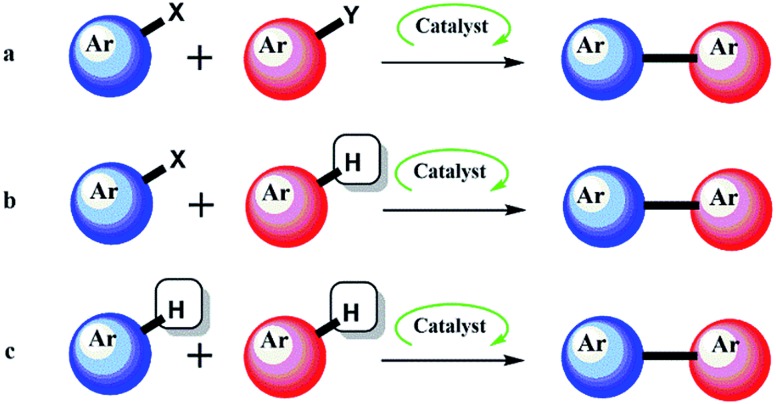
Aryl–Aryl bond formation using traditional catalysis (a), and direct arylation by one (b), and two (c), C–H activation events.

Aryl–aryl (Ar–Ar) bond formation and the heteroaryl analogues (Ar-Het and Het–Het) are undoubtedly one of the most important transformations in organic chemistry, and compounds containing the Ar–Ar, Ar-Het and Het–Het moieties are ubiquitous within the synthesis and pharmaceutical industry.^1^ Examples include Crestor, Celebrex and Diovan, representing sales of $5.2 billion, $2.2 billion and $2.1 billion respectively in 2013.^2^ Lipitor, which contains two Ar-Het bonds is the best-selling drug of all time (∼$125 billion). Classical methods for the creation of these bonds include well-known transformations such as the Suzuki–Miyaura, Stille, Negishi and other named reactions.^3^ The importance attributed to the discovery of these reactions was recognised in 2010 by the award of the Nobel prize in Chemistry to Suzuki, Negishi (and Heck for alkenyl variants) for ‘palladium-catalyzed cross couplings in organic synthesis’.^4^ These transformations usually include the reaction of organometallics species involving B, Sn, Si, Zn, Mg *etc.* and a wide range of aryl halides or halide equivalents in the presence of a transition metal (Fig. 1a). While high yields and selectivities can now be obtained by these traditional methods, they still suffer from major drawbacks. Firstly, both coupling partners must be preactivated, which is inherently wasteful since it necessitates the installation and subsequent disposal of stoichiometric activating agents. The installation of the activating group(s) itself is not trivial, often requiring several steps and suffering from the usual regioselectivity problems, as well as the secondary issues of generating waste from reagents, solvents and purifications. Recently, there has been a growing appreciation by the pharmaceutical industry of the costly environmental and economic impacts of many traditional organic syntheses.

Modern methods to synthesise Ar–Ar compounds and the heteroaryl analogues *via* C–H activation^5^ are among the published ‘Wanted List’ of top pharmaceutical companies.^6^ A modern, efficient and environmentally friendly method for the formation of these compounds is termed Direct Arylation (DA).^7–9^ DA by catalytic C–H activation (Fig. 1b and c) is a more convenient process, because it avoids the preactivation steps. Using DA strategy, a myriad of reaction pathways that can surpass more established routes in terms of atom economy, environmental impact and cost have emerged. Indeed, the versatility of DA has allowed its application in many areas of chemistry beyond simple substrates.^10^

### Heterogeneous catalysis

Hetereogeneous catalysts (especially supported catalysts) function in a number of core businesses from energy production in fuel cells, to crude oil refinement to the reduction of harmful car exhaust emissions. Any discussion on heterogeneous catalysis requires at least a brief discussion on its definition. For a full discussion the reader is directed to an early review^11^ on heterogeneous catalysis and an excellent and insightful discussion by Crabtree.^12^ As early as 1901, Oswald classified catalysis into four categories.^13^ However, the once sharp distinction between these areas has become vague over the last few decades. More specifically, the distinction between homo- and heterogeneous catalysis, once considered clear, has merged in recent years by work on clustered, metal nanoparticles and nanomaterials.^14,15^ In fact, both Schwartz^16^ and Crabtree^12^ have commented on the unsatisfactory usage of heterogeneous/homogeneous nomenclature in the discussion of catalysis. Crabtree prefers the use of homotopic and heterotopic. This terminology refers to the *site* of catalysis rather than the *phase* of catalysis and allows for the mechanistic distinction between a catalyst with a ‘cocktail’ of sites available (heterotopic) and that with a single site of catalysis (homotopic). For example Pd/C, which would have multiple sites available for catalysis, would be deemed heterotopic. Similarly, a *soluble* nanoparticulate, which would be operationally homogeneous, would nevertheless be deemed heterotopic. The term homotopic is reserved for catalysts with a single type of site, irrespective of solubility. These terms appear to create a clear distinction and could be used in the context of detailed mechanistic discussion. However, the nomenclature is not without shortcomings. For example, Crabtree's terminology supposes that only a single component (*i.e.* atom) of a transition metal complex constitutes the active site, whereas ligands of course play a crucial role in modifying both electronic and geometric properties of their coordinated metal. They can also directly activate substrates, and in this sense can themselves be argued as active components. Thus all moieties, except isolated metal atoms, must be considered heterotopic.^17^

That said, the goal of this perspective is to provide an entry point for academics and industrialists, and to bringing readers up to speed with DA in the context of homo/heterogeneous catalysis with a view towards providing a platform for the design of new catalysts with key properties (*e.g.* recyclability). Thus the phase-driven terminology homo- and heterogeneous suffice here.

Many reports exist which tackle the topic of heterogeneous *versus* homogeneous catalysis in the context of cross-coupling reactions and the difficulty in their definitive assignment.^18–21^ Indeed, well-designed and executed experiments have been insightful, but have also resulted in contrasting conclusions.^22–25^ One could attempt to theoretically distinguish between heterogeneous and homogeneous catalysis on the basis of phases (suspension or solution) but this is not trivial as will be discussed later. Practically, the difficulty in distinguishing between the two forms is complicated by situations where a small amount of soluble homogeneous catalyst particulates to a highly active insoluble heterogeneous catalyst, and likewise where some of an insoluble supposed-heterogeneous catalyst dissolves to form an active soluble homogeneous catalyst. There are added complications such as dissolution and reattachment^26^ and of course many homogeneous catalysts decompose at some point leading to time-dependent behaviour. A situation whereby a soluble colloid or small nanoparticle catalyses a reaction *at its surface*, perhaps leads to the most difficult type to ascertain and herein the term heterotopic fits well as previous discussed. These uncertainties have undoubtedly hampered the judicious design of heterogeneous catalysts for cross-couplings but have also plagued other areas of transition-metal catalysis such as biomass conversion.^27^ However, it is still possible to discriminate (to a certain extent) between a homogeneous and heterogeneous catalytic systems in practice, or at least when it comes to the design and operation of a reaction.

In comparison with heterogeneous catalysis, homogeneous catalysis usually allows for reliable reproducibility and scale up (certainly in cross-couplings, often in hydrogenations but less so in other areas: *e.g.* catalysts for *e.g.* ammonia synthesis). It also provides wide opportunity for optimisation especially in terms of introducing and optimising defined ligands. However, homogeneous catalysis suffers from a number of drawbacks. For example, separation of the expensive ‘catalyst’ from the product for re-use is very difficult and usually not possible. Also homogeneous catalysts tend to lose their catalytic activity because of metal aggregation and precipitation.^28^ The latter is a major issue for the pharmaceutical industry^29^ and is of particular environmental and economic concern in large-scale syntheses. Heterogeneous catalysis is an attractive solution because the catalysts involved possess good thermal stability and can usually be removed from the reaction mixture and recycled.^30^ While true heterogeneous catalysis has been the workhorse of important organic synthetic reactions (such as hydrogenations). It has found less use in cross-coupling reactions. If we take the Suzuki–Miyaura reaction (which in its most common form involves the transition-metal catalysed coupling of boronic acids and aryl halides), it has been the development to unactivated substrates, sterically hindered substrates and the use of milder conditions which has dominated the research field. In contrast, relatively scant research has been reported on developing more active and selective heterogeneous catalysts to carry out Suzuki–Miyaura couplings.^31^ Even fewer reports have emerged on the analogous DA reactions. However, recent progress has been made in the investigation of heterogeneous systems for DA. Palladium has been the most employed transition-metal,^32^ but a few reports have appeared which use other transition metals.

## Examples of direct arylation mediated by homogenous and heterogeneous *catalysis* using heterogeneous-type *catalysts*

**Fig. 2:**
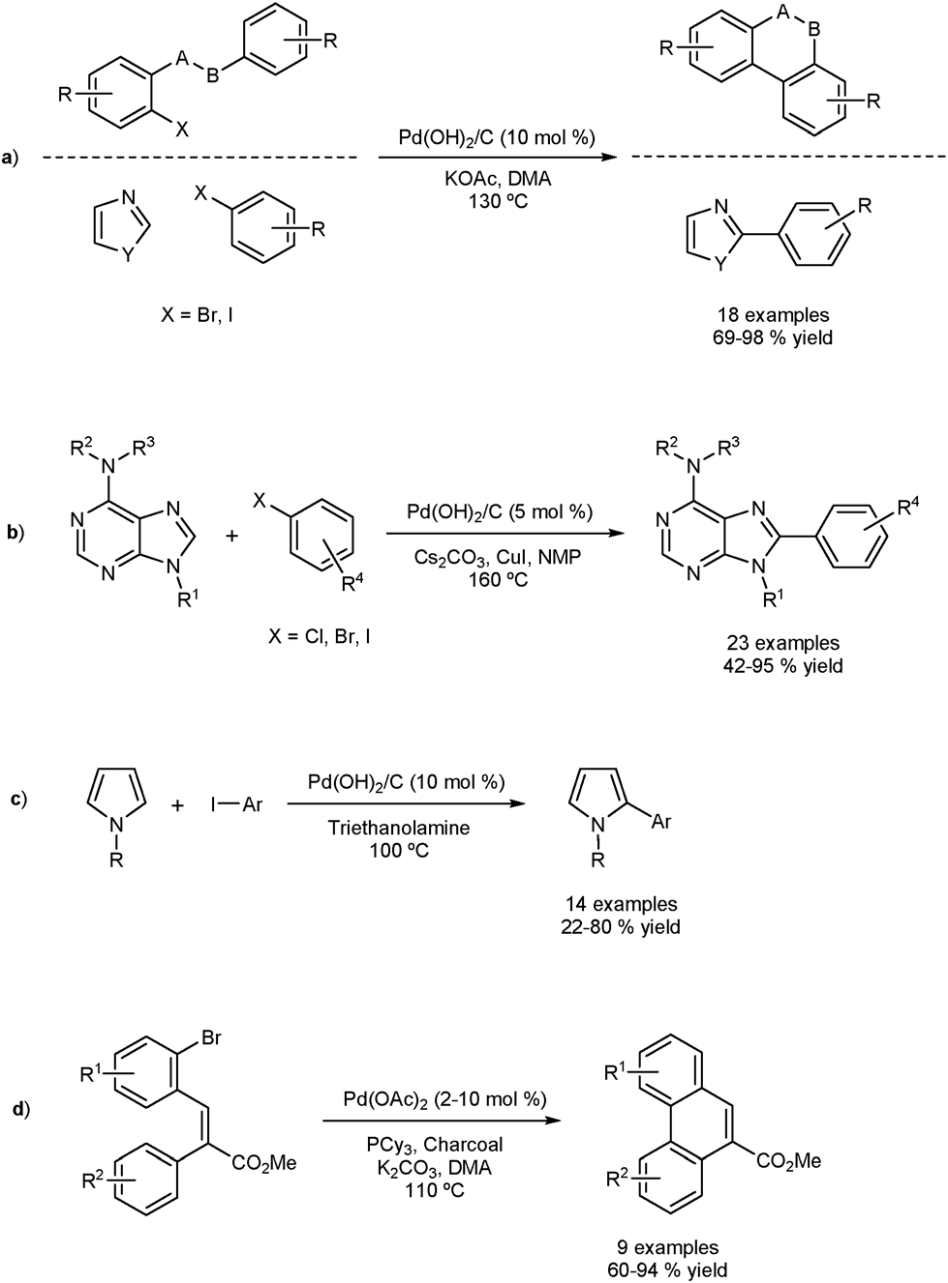
Direct arylation using heterogeneous catalysts with evidence of homogeneous catalysis (a), (b). Pearlman’s catalyst applied to the direct arylation of pyrroles (c). Strategy to adsorb unstable palladium particles generated during intramolecular direct arylation (d).

The Fagnou group reported the DA of aryl iodides and bromides using a traditionally-heterogeneous catalyst Pd(OH)_2_/C (Pearlman's catalysts) as shown in Fig. 2a.^33^ Intra- and intermolecular DA were achieved in very good yield. Two approaches were used based on the three-phase test concept to determine homo- or heterogeneity. The three-phase test involves anchoring one of the coupling partners onto a solid support, and a supposed non-soluble catalyst. No cross-coupling should be observed if the process is completely heterogeneous.

Firstly, a resin-bound substrate underwent coupling, proving interaction between the matrix bound catalyst and the supported substrate. Secondly, it was found that a silica/thiol based Pd scavenger sequestered the reaction. These results confirm that under the reaction conditions, leaching occurs and homogeneous catalysis is likely. The scope of the catalyst was extended by others in a number of interesting direct arylation processes (Fig. 2b and c).^34–36^ Felpin and co-workers formed Pd(0)/C *in situ* by using charcoal and Pd(OAc)_2_ and applied the catalyst in DAs.^37^ The presence of charcoal is thought to act as a stabilizer for active palladium species and as a sponge for inactive ones. The reaction also proceeds in the absence of charcoal, suggesting homogeneity. This method allowed the preparation of phenanthrenes and naphthoxindoles using DA as part of a three-step sequence (Fig. 2d).

**Fig. 3:**
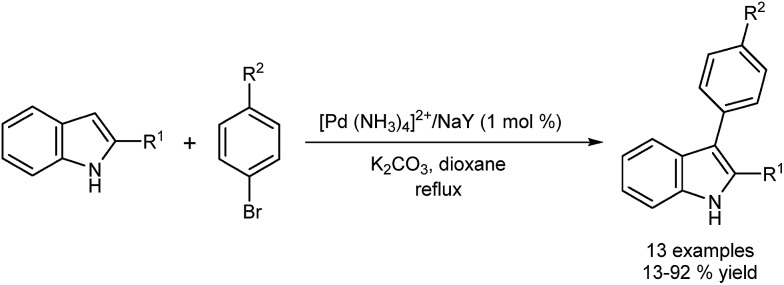
Palladium supported on zeolite applied to the C3 arylation of indoles.

Djakovitch *et al.* reported DA at the 3-position of 2-indoles using Pd supported on a zeolite, and Pd on silica as catalysts.^38^ The authors term these ‘heterogeneous palladium catalysts’. Analysis of the two systems revealed differences in the nature of the catalyst involved in each transformation. The first palladium catalyst (Pd supported on zeolite) maintained activity after a hot filtration which suggested the presence of active soluble catalysts. The activity of the second catalyst tested (Pd supported on mesoporous silica) was lost after the hot filtration revealing the potential for heterogeneous catalysis. In later work (Fig. 3), a study involving the DA of 2-substituted indoles was extended using a zeolite-based catalyst.^39^

**Fig. 4:**
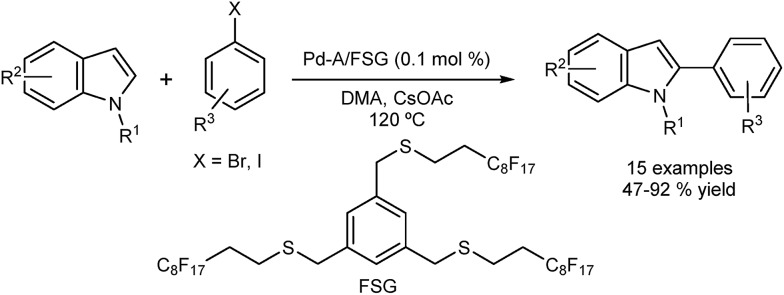
Palladium supported on fluorous silica gel for the direct C2 arylation of indoles.

Cai and co-workers reported selective direct C2 arylation of indoles employing palladium immobilized on fluorous silica gel (FSG) (Fig. 4).^40^ The catalyst was reused in seven successive runs showing a 20% loss in activity. A hot filtration test revealed loss of activity, which suggests a catalyst of heterogeneous nature. The possibility that the leached palladium was re-deposited onto the support should also be considered.

**Fig. 5:**
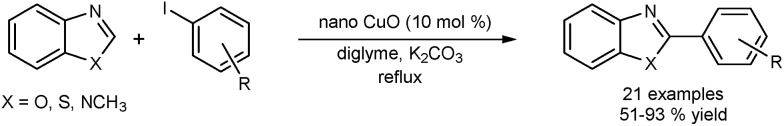
CuO nanospindles used as catalysts for the direct arylation of heterocycles.

Zhang and co-workers reported the use of CuO nanospindles to perform the direct arylation of heterocycles such as benzoxazole, benzothiazole and 1-methylbenzimidazole with aryl iodides (Fig. 5).^41^ The catalyst was recycled in 3 successive runs without significant loss of activity. X-ray diffraction (XRD) analysis of the catalyst after the reaction confirmed retention of crystallinity. Copper leaching was determined using atomic-absorption spectroscopy (AAS). The catalyst was separated from the reaction media by centrifugation and the leached species did not show any activity. The authors believe that the reaction occurred on the surface of the CuO and that any leaching was of a catalytically inactive copper species. Again it is difficult to rule out a re-deposition mechanism. The scope of the catalyst was extended to the use of aryl bromides.^42^

**Fig. 6:**
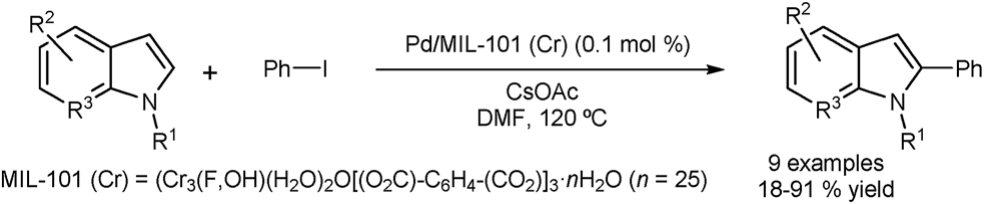
Palladium nanoparticles encapsulated in a metal-organic framework for C2 direct arylation of indoles.

Cao and co-workers reported the DA of N-substituted indoles using Pd nanoparticles encapsulated in a metal–organic framework (MOF) incorporating Cr.^43^ The arylation occurs selectively at the C2 position, with almost no formation of the corresponding C3 arylated indoles (Fig. 6). The catalyst could be recovered by centrifugation and was tested in five consecutive runs with only minor loss in activity. Metal leaching was examined using Inductively Coupled Plasma (ICP) and showed a low level of Pd leaching (0.4 ppm) and no Cr leaching. Characterisation of the catalyst before and after the reaction appeared to confirm the stability of the catalyst structure.

**Fig. 7:**
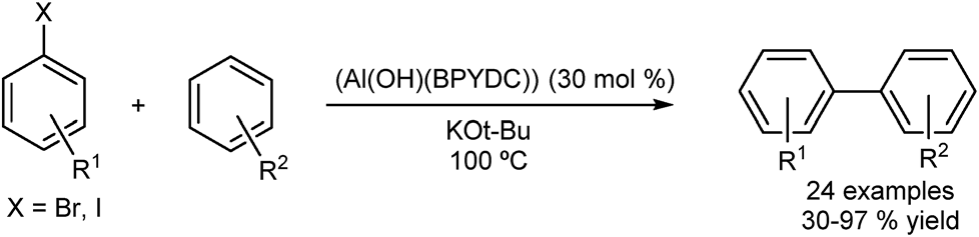
An apparent transition metal-free direct arylation of arenes.

An interesting transition-metal-free system was reported by Jiang and co-workers.^44^ Care must be taken when describing such reactions as transition metal-free. It has been shown that trace (ppb level) available in the inorganic bases used can turnover cross-coupling reactions.^45^ Jiang described an aluminium-based MOF (Al(OH)(BPYDC)) used in the DA of arenes with aryl iodides and bromides (Fig. 7). The π,π-stacking and ion–π interactions between the metal–organic framework, the potassium ion and the arenes apparently allow the direct arylation in absence of transition metals.

**Fig. 8:**
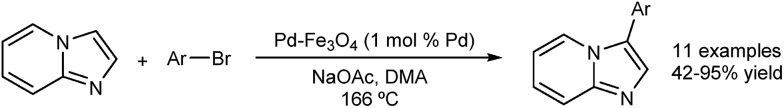
Pd–Fe_3_O_4_ nanoparticles applied to the C–H arylation of imidazo[1,2-*a*]pyridine.

The catalyst was reused in five runs without losing activity and the stability of the catalyst was confirmed by X-ray diffraction (XRD) after the reaction. The heterogeneity of the catalyst was also indicated by a hot-filtration test. Kim and co-workers described a magnetically recoverable catalyst for the direct arylation of imidazo[1,2-*a*]pyridines with aryl bromides (Fig. 8).^46^ The catalyst was reused over 10 runs and high yields (>80%) were maintained. However the authors did not carry out any experiments to determine homo- or heterogeneity and while the recyclability of the catalyst seems to point to a heterogeneous nature, homogeneous catalysis mediated by trace leaching of highly active, small particles is likely at these temperatures.

**Fig. 9:**
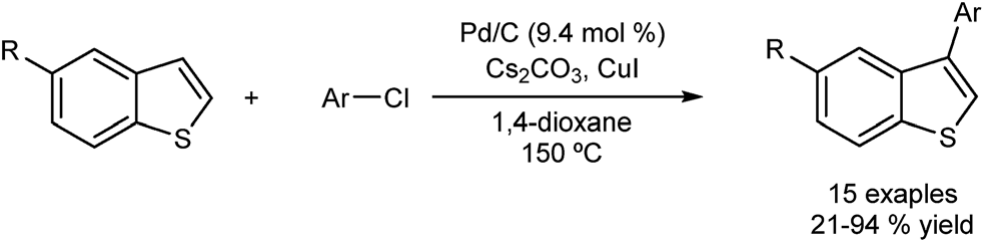
Pd/C as catalyst for the direct C–H functionalization of benzo[*b*]thiophenes.

The Glorius group have reported the direct arylation of benzo[*b*]thiopenes with aryl chlorides using Pd/C as catalyst.^47^ The system is ligand-free, insensitive to air and moisture, and it provides the corresponding arylated products with complete selectivity at the C3 position (Fig. 9). Unfortunately the group has not found an efficient method to recycle the catalyst, although they have performed several experiments which provide evidence of the heterogeneous nature of the catalyst, including poisoning, hot-filtration and three-phase tests. The presence of leaching (<4 ppm) was established by X-ray fluorescence (XRF), but a hot-filtration test revealed the inactivity of this leached species. Also the three-phase and poisoning tests seem to discard a fast re-deposition of homogeneous species onto the support.

**Fig. 10:**
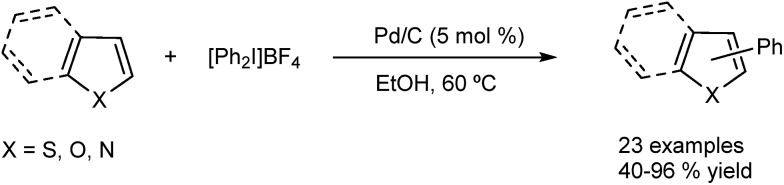
Pd/C as catalyst for regioselective C–H functionalization of thiophenes.

The same group has extended the use of the Pd/C catalytic system to the arylation of other heterocycles such as thiophenes, indoles, furans and benzofurans.^48^ In these systems, iodonium salts were used instead of halides as the arylating agents (Fig. 10). Recently the methodology has also been applied to other arenes in good yields and regioselectivity.^49^

**Fig. 11:**
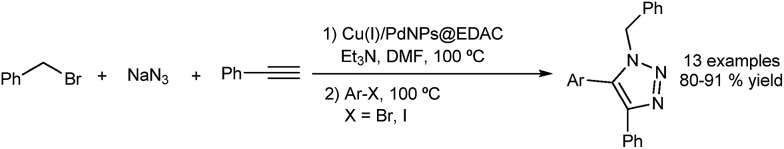
Cu(I) and Pd(0) supported on ethylenediamine-functionalized cellulose (EDAC) as catalyst for a 1,3-dipolar cycloaddition/direct arylation sequence.

Shaabani's group have established an apparently heterogeneous system to prepare trisubstituted triazoles through a 1,3-dipolar cycloaddition/direct arylation sequence (Fig. 11).^50^ For this purpose they have supported copper(i) and palladium(0) on ethylenediamine-functionalized cellulose. The catalyst could be reused several times using a filtration step with only minor decreases in activity. However, again a mode of action involving successive leaching of small, active catalytic species would not have been identified by the filtration and recycling tests.

**Fig. 12:**
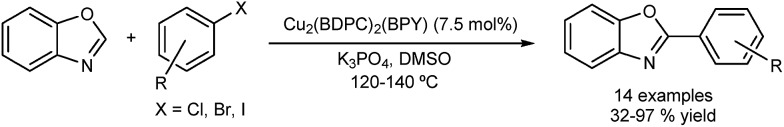
Cu metal-organic framework for the direct arylation of benzoxazole.

Phan and co-workers used MOF Cu_2_(BPDC)_2_(BPY), for the DA of benzoxazole using aryl halides (Fig. 12).^51^ The group also prepared a nickel-MOF catalyst which was also used for the DA of benzoxazole, this time using boronic acids.^52^ Both catalysts were recovered by filtration and reused in several runs. Yields of over 80% were maintained. Again, a filtration test was performed for both Cu and Ni-based catalysts and no activity was observed in the filtrate.

## Discussion & outlook

Heterogeneous catalysis is a well-developed and mature aspect of chemical synthesis. While traditionally-heterogeneous catalysts have been used in cross-coupling reactions, in most cases the active catalyst is of a homogeneous nature (in most Suzuki–Miyaura reactions and almost exclusively in Mizoroki–Heck reactions). When it comes to Direct Arylation (DA), studies using heterogeneous catalysts have only emerged in the last couple of years and few have shown good evidence for heterogeneous catalysis. It those that have shown promise, only basic tests for homo-/heterogeneity have been carried out. No experiments to elucidate homo-/heterotopicity, no *in situ* spectroscopic techniques have been used^53^ and only a few kinetic studies have been reported.^47–49^

When it comes to distinguishing between homo- and heterogeneous, the question arises: does it matter? The answer is yes, but perhaps less so if your goal is simply to access a synthetic target in an academic research setting. That said, catalytic variables of interest to all (selectivity, activity, life-time *etc.*) depend on the precise nature of the kinetically dominant catalyst. In an industrial setting the nature of catalysis is crucial. More often than not, heterogeneous catalysis will leave lower levels of residual metal after work up, and is more likely to allow for recyclability and flow technology. Easy isolation, reuse or continuous use of a stable catalyst are key goals of many chemical processes, particularly in the preparation of active pharmaceutical ingredients (APIs), where the fate of the metal catalyst and ancillary ligands (phosphines for example) is of particular importance. Finally and most importantly, gleaning an insight into the nature of catalysis, will be key to the future design of catalysts with favourable properties. Even in terms of catalysts which are clearly heterogeneous in nature, much exploration is needed. It is now well-accepted that heterogeneous particles are in fact fluid entities, and that morphing of particles and their surfaces, in response to the plethora of omnipresent stimuli present in a reaction, is quite general and surface changes such as reorganisation of particles in response to physical or chemical inducements, results in changes of surface.

While protocols such as the three-phase technique, poisoning, hot-filtration, recyclability *etc.* provide some insight into the nature of the catalytic media, there are numerous flaws with each technique.^18,19^ Ensemble averaged measurements (post-reaction Transmission Electron Microscopy TEM, ICP) which allow one to ‘view’ a catalyst pre- and post-reaction, cannot capture events in real time. Thus *in situ* techniques which minimally interfere with the reaction are crucial for understanding the properties of catalysts. Theoretically, *in situ* techniques (*e.g.* various types of *in situ* spectroscopy) allow establishment of the structure and composition of compounds formed in different stages of a catalytic reaction. In the last few years developments in a variety of areas have resulted in a number of advanced, direct and *in situ* techniques to evaluate the internal structure of the catalyst and the surface features.^26,54–58^ In some of these cases involving Sonogashira reactions, surface atom dissolution is not possible and thus pure surface catalysis is in operation. Environmental TEM (E-TEM) has evolved, and in some cases allows for atomic scale information about the nature and distribution of metallic particles, and *in situ* characterization of catalyst surfaces with atomic resolution. However, the technique is still limited by the difficulty in its operation under certain reaction conditions. Despite the numerous other X-ray and spectroscopic imaging methods available for *in situ* analysis (vibrational spectroscopy, electronic spectroscopy, X-ray diffraction and X-ray spectroscopy methods), we are not aware of any detailed investigation of DA using real time *in situ* surface analysis. Thus we have chosen to highlight two of the most powerful current techniques, which should not only find useful application in the evaluation of nanocatalysts, but could provide far reaching and detailed information about the location of catalysis on the nanoparticle surface.

**Fig. 13:**
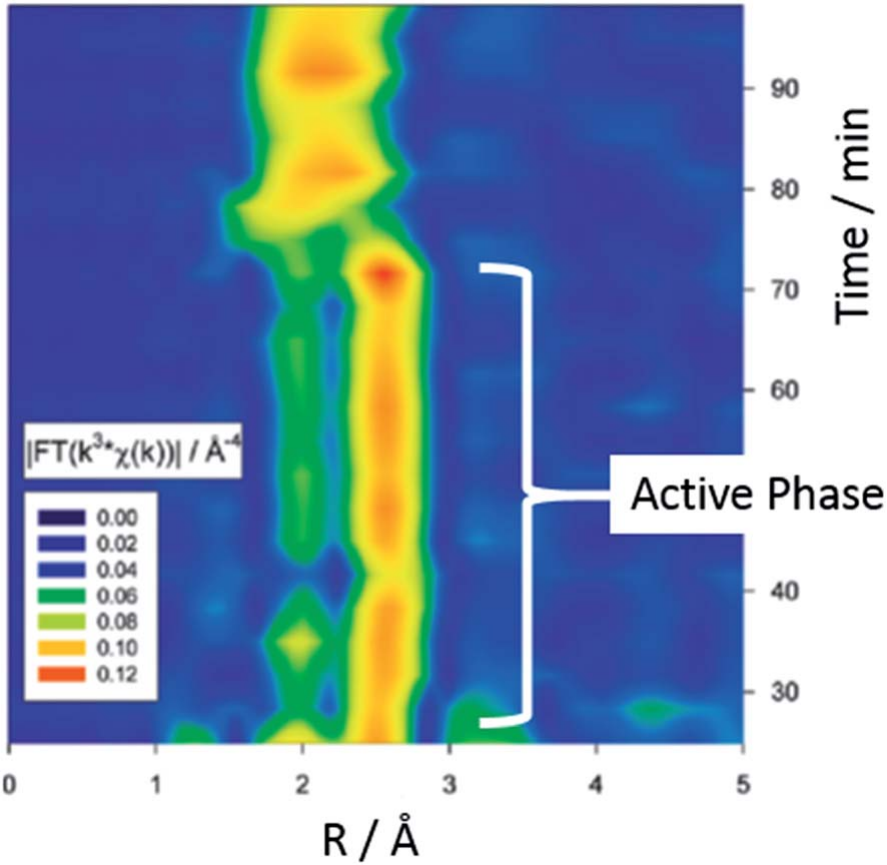
Recent use of *in situ* techniques which could be applicable to direct arylation. Time-dependent Fourier-transformed EXAFS spectra of the liquid phase in a Heck reaction of styrene and bromobenzene (adapted with permission from (*J. Am. Chem. Soc.*, 2011, **133**, 3921). Copyright (2011) American Chemical Society).

Extended X-ray absorption fine structure EXAFS (and its energy dispersive and quick scanning variant) are excellent methods for the analysis of isolated metal atoms *e.g.* surface organometallic species and very small metal particles (<100 atoms). In this technique, X-rays (of a narrow energy resolution) are irradiated on a sample and the incident and transmitted X-ray intensity are recorded as a function of initial X-ray wavelength. An absorption edge is observed when an incident X-ray energy matches the binding energy of an electron, and each set of edges is representative of an atom's chemical environment. These techniques allowed Reimann *et al.* to follow the leaching of Pd from a heterogeneous catalyst *in situ*.^59^ The group conclude that colloids of Pd(0) present during the active phase of catalysis are transformed into the catalytically active species (‘molecular’ Pd) in a rate determining step. Fig. 13 shows a Fourier-transformed EXAFS spectra of the liquid phase. Two well-separated signals at *ca.* 1.8 and 2.5 Å correspond to the active phase involving Pd colloids with a size of *ca.* 2 nm. The broad signal at 2.0 Å was well fitted to bromo-palladates such as [Pd_2_Br_6_]^2–^ or [PdBr_4_]^2–^ which showed increased formation as the reaction rate decreased.

A similar investigation is tempting in the context of DA reactions given the similarity of reaction condition for both systems. It should be noted that XAS is of course an averaging technique, and hence even the observation of a clear change in the average chemical environment about a transition metal during a reaction does not prove that such a change creates a catalytically relevant active species – unless the rate of emergence of this new chemical environment precisely matches the reaction kinetics (*vide infra*).

**Fig. 14:**
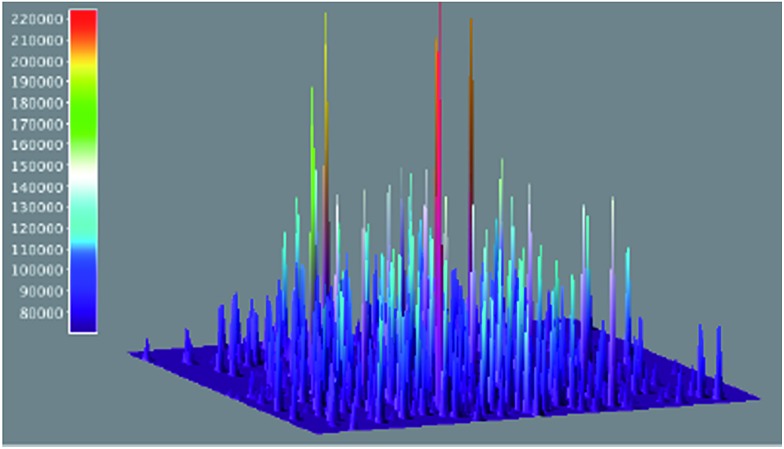
3-D accumulated bright events (500 frames), of azoles formed *via* click cycloaddition of an alkyne and an azide on copper nanoparticles, the area corresponds to 80 μm (adapted with permission from (*Nat. Commun.*, 2014, **5**, 4612). Copyright (2014) Nature Publishing).

The use of single-molecule fluorescence spectroscopy (SMFS) has tremendous potential to glean an insight into the location of active catalytic sites. Here a laser beam is used to excite molecules and the resulting emission spectra is recorded by a detector. Probe molecules are necessary if the reaction being investigated does not involve fluorescent or fluorogenic molecules. Only very recently has this technique been used for the study of heterogeneous cross-coupling. Decan *et al.* investigated the reaction of an azide with an alkyne in a Cu-catalysed click reaction to form the corresponding triazole product.^60^ Fig. 14 shows 3-D accumulated bright events (500 frames), of an azole formed *via* click cycloaddition of an alkyne and an azide on copper nanoparticles. Single-molecule studies reveal that the reaction proceeds through initial formation of Cu-acetylides on the nanoparticle surface followed by azide attack. Under the reaction conditions used, only one catalytic event takes place per nanoparticle at a given time and observed systematic repetition of ‘bursting’ locations confirmed that the reaction likely takes place at the Cu nanoparticle surface and not on leached copper. Intriguing details, such as the product triazole displaying a residence time of about 3 s at the Cu surface following the reaction, also emerged from the study. Because the method operates at ambient temperature and pressure and in a condensed phase, it can be applied to the growing number of liquid-phase industrial organic transformations. The use of SMFS in direct arylation reactions could involve monitoring the disappearance of astutely-designed fluorescent substrate and the appearance of a fluorescent product. A wide field microscope should then be able to map the spatial distribution of catalytic activity over an entire nanocrystal. Although quite a way off, correlation of specific activity (at different faces, edges, corners *etc.*) to localize catalytic activity and observation of leaching from crystal surfaces would provide invaluable information on the catalytic events. Additional features of this approach would be the ability to time-stamp a transition metal mediated transformation by the use of two different fluorescent indicators. Despite all these analytical advances, very little is still known about the conduct of catalysts *in situ* and at the molecular level, but over the next few years we expect a steady development in technology allowing the operando spectroscopy study of heterogeneous catalysts. Additionally, we expect the top-down spectroscopic approaches discussed above to unite with bottom up theoretical studies^61^ (not discussed here).

However, even reliable inference on the formation of one or another compound in the course of the reaction gives no information about the role of this compound in the catalysis. For example if we consider catalyst leaching when using heterogeneous, catalyst-precursors. The observation of the leached material is clearly a key step in proving a homogeneous mechanism of catalysis. However, leaching can only be considered as proof of a homogeneous mechanism if the entire catalyst (initially present in the solid state) has passed into the truly dissolved state. Despite this key point, none of the publications devoted to heterogeneous catalysts, in even traditional cross-coupling reactions, report on the complete leaching of palladium from heterogeneous precursors. It is likely that in most cases both dissolved and solid forms of the catalyst coexist during the course of the reaction, and consequently, the observed catalytic activity cannot be attributed only to the dissolved form.^20^ Having no additional information on the role of dissolved (pre)catalyst in catalysis, it is impossible to establish whether leaching is 1. A process of the deactivation of the functioning catalyst, or 2. A process required to activate (by dissolution) a heterogeneous precatalyst. A similar problem arises when colloidal particles of the catalyst formed from homogeneous precatalysts are detected. The coexistence of truly dissolved molecular compounds and solubilised colloidal particles of the catalyst obstructs the determination of the true catalyst. Moreover, if both forms exert catalytic activity, it becomes almost impossible to evaluate the contribution of each towards product formation.

Kinetic studies are the only tool to determine the catalytic role of substances which are experimentally observed by *in situ* techniques. In fact “catalysis is, by definition, purely a kinetic phenomenon”.^62^ Even basic kinetic measurements, based exclusively on data derived from conversion of starting materials, or accumulation of products, can enable the calculation of integral and differential values of catalytic activity and selectivity. Thus application of *ex situ* methods together with kinetic data allows for valid inferences about the type of catalysis. For example, Reetz and Westermann,^63^ having found the formation of colloidal palladium particles in Mizoroki–Heck and Suzuki–Miyaura reactions, examined a series of aliquots taken throughout the reaction. Because nanoparticles were detected only in aliquots taken during the period of catalytic turnover, it is likely that the particles represent active species and that catalysis takes place on the surface of the colloidal metal particles. Although a fast, subsequent dissolution to soluble molecular species is difficult to rule out. However, such sampling methods also run into insuperable problems, since data on the structure and reactivity of intermediates (indispensable for a full comprehension of the reaction) are often inaccessible. Simultaneous measurement of catalytic activity and *in situ* time-resolved analytical techniques provides additional possibilities for studying a working catalyst. Such an approach can be referred to as the true operando study of catalysis. Here, identification of intermediates by *in situ* techniques under truly catalytic conditions, in combination with elucidation of product concentration-changes, allows for good time-resolved catalytic activity profiles. These can then be used to provide hypotheses on the role of one or another species in catalysis.

Catalyst selectivity is the ability of a catalyst to favour the desirable reaction rather than the undesirable reactions. On the other hand, the *differential selectivity* of a catalyst towards the desired product is often understood as the ratio of the rate of its formation to the sum of the formation rates of all the reactions products. The selectivity of a catalyst is rarely considered^64,65^ in kinetic investigation of cross-coupling reactions. However, selectivity and, particularly, differential selectivity measurements have a number of advantages in comparison with catalytic activity measurements.^64^ Differential selectivity measurements allow one to elucidate the fine details of mechanisms in reactions with complex product compositions and those involving catalytic transformations outside the catalytic cycle (*e.g.* formation, deactivation and/or poisoning of the active species). The main advantage of differential selectivity in comparison with catalytic activity protocols, is the former's independence of the total amount of truly active species. In the types of reactions discussed herein, the amount of true catalyst is unknown in most cases, especially when its nature is obscure. In addition, active species are generally highly reactive substances in ultra-low concentration (because of their deactivation and/or poisoning). Moreover, the low concentration creates problems of experimental detection and precise measurements of the active species. These circumstances hamper the correct calculations of catalytic activity because the value taken as the total amount of the active catalyst, is actually the amount of the catalyst precursor loaded in a reactor.

However, using differential selectivity methodology, it is possible to study reactions carried out with normal *loaded* substrate/catalyst ratios, in contrast to the usual studies, which only study the (small) amount of *working catalyst*. This avoids incorrect conclusions based on non-realistic model studies.^66^ Unlike the case with catalytic activity, the change in differential selectivity in discriminatory experiments can be unambiguously attributed to a change in the nature of the active species. Thus, measurements of differential selectivity are best suited for operando investigations using *in situ* techniques for observation of the working catalyst.

Differential selectivity studies have elucidated important information regarding the active species in the two most studied reactions: Mizoroki–Heck and Suzuki–Miyaura.^64^ As discussed earlier, the complexity of these studies is due to the fact that palladium compounds used as catalyst precursors are converted to several, potentially active, interconverting forms. For competitive reactions of two substrates that convert into two products, it is possible to draw the dependences of one product yield *versus* another to obtain the so-called phase trajectories of reaction. These phase trajectories allow for a qualitative estimation of differential selectivity. The shape of the phase trajectory, determined by the kinetics of product accumulation, is a very specific and sensitive parameter. A discrepancy in the phase trajectories obtained by varying the catalyst precursor or adding some additives to the reaction mixture unambiguously indicates a change in the nature of the catalytically active species making a major contribution to substrate conversion. In the same way, overlapping phase trajectories indicate that the nature of the catalyst remains the same.

**Fig. 15:**
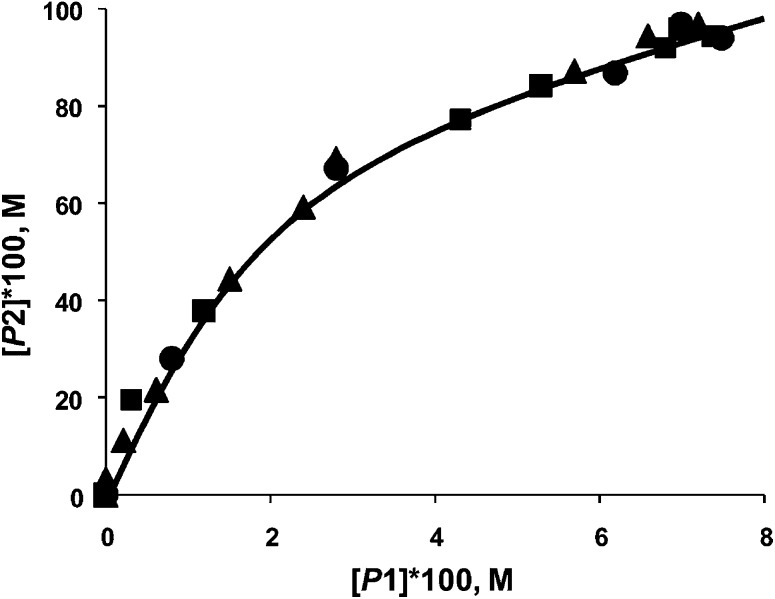
Phase trajectories of Mizoroki–Heck reaction of competing aryl bromides (bromobenzene and 4-bromoacetophenone) with styrene using PdCl_2_ (), Pd/Al_2_O_3_ (■) and Pd/SiO_2_ (▲) as catalyst precursors, where [P1] – [stilbene] and [P2] – [4-acetylstilbene].^64^

**Fig. 16:**
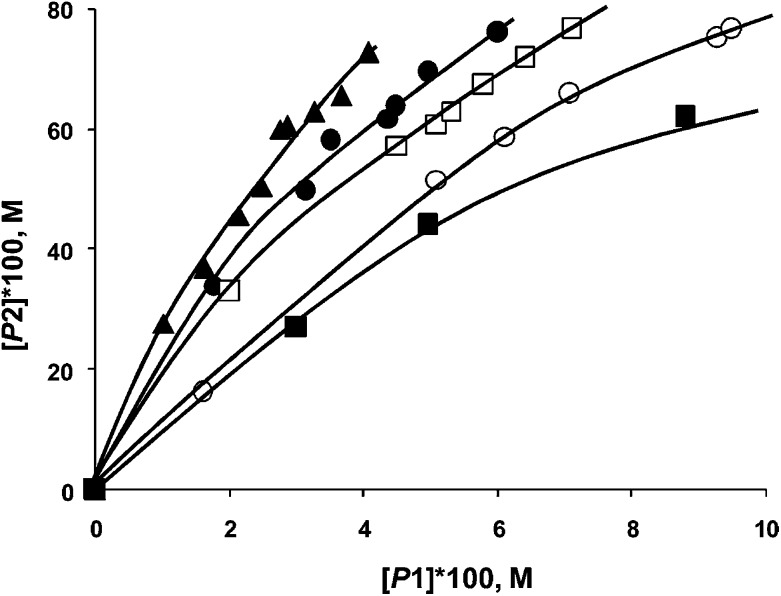
Phase trajectories of Suzuki–Miyaura reaction of competing aryl bromides (bromobenzene and 4-bromoacetophenone) with phenylboronic acid using PdCl_2_ (■), Pd/C (▲), Pd/SiO_2_ (), Pd/MgO (□), and Pd/Al_2_O_3_ (○) as catalyst precursors, where [P1] – [biphenyl] and [P2] – [4-acetylbiphenyl].^64^

In the Mizoroki–Heck reaction it was established that with soluble (*e.g.* PdCl_2_) and insoluble (Pd/Al_2_O_3_) catalyst precursors, the phase trajectories completely overlapped (Fig. 15) when 4-bromoacetophenone and bromobenzene are used as competing substrates with styrene.^67^ The same situation occurred when aryl iodides were used, or with a combination of aryl iodide and aryl bromide. This is convincing evidence for the formation of the same active species from a soluble and insoluble catalyst precursor, and allowed the authors to infer homogeneity. In the case of the Suzuki–Miyaura reaction, two different situations have been observed. In one case, the phase trajectories using reactive aryl iodides as competing substrates with varying catalyst precursors (soluble and insoluble) overlapped very well (not shown). Aryl iodides lead to a more efficient dissolution of Pd and a homogeneous mechanism is more probable. However the phase trajectories using aryl bromides with soluble or insoluble catalyst precursors were substantially different (Fig. 16). Thus it can be concluded that the active species in both is different, and that at least a contribution from heterogeneous catalysis is likely.

The study of the kinetics of DA reactions is expected in the near future and we would advocate the use of the differential selectivity approach. It is also hoped that leaders of the field will start to utilize *in situ* techniques in combination with kinetic based approaches. Common palladium-leaching-aids such as boronic acids and some bases are absent in DA reactions, thus new discoveries on the behaviour of nanocatalysts are expected. The usual demands of a new methodology (increased substrate scope and late stage application to natural product synthesis) will trundle on in parallel, and the use of heterogeneous catalysis for double C–H activation (Fig. 1c) is surely not too distant.

As improvements are made in terms of *in situ* time and space resolution in the liquid phase, we can begin to correlate spatial activity patterns to chemical selectivity and reactivity. Thus the judicious design of catalytic materials with specific architecture and distribution of active sites can be realised. Ultimately, we believe that only at the confluence of the disciples of organic chemistry, chemical kinetics, catalysis, surface chemistry and spectroscopy, each burdened and empowered with their own histories and experience, will the most exciting developments be made.
